# Influence of Shopping Bags Carrying on Human Responses While Walking

**DOI:** 10.1155/2018/5340592

**Published:** 2018-06-27

**Authors:** Mohamed Z. Ramadan, Tamer M. Khalaf, Adham M. Ragab, AbdElatty A. AbdElgawad

**Affiliations:** ^1^Department of Industrial Engineering, College of Engineering, King Saud University, Riyadh, Saudi Arabia; ^2^Department of Mechanical Engineering, College of Engineering, Al-Azhar University, Cairo, Egypt

## Abstract

Shopping as a daily activity that involves carrying shopping bags in hands might be associated with risk factors contributing to the development of low back pain (LBP) and strains and sprains in the upper extremity. A three-way repeated measures experiment was conducted for the purpose of the study. The independent variables were holding style, carrying technique, and shopping bags' weights. The dependent variables were cardiac cost, muscles' activities as a percentage of their maximum voluntary contraction's EMG (%MVC), peak plantar pressure (PPP), and discomfort rating. Carrying grocery bags with both hands to the sides of the body using shopping bags' holder was favorable and advantageous to other carrying conditions in terms of less cardiac cost, less %MVC, less peak plantar pressure, and less discomfort. It is useful to carry grocery bags close to the body with both hands using holders that are available in the local market.

## 1. Introduction

Shopping is one of the typical daily activities for a broad range of variable populations of human beings. It is almost undeniable that shopping is a daily requirement for a considerable portion of the population, where shoppers carry and transport their items such as groceries, clothing, and other goods in flexible plastic bags. Whether it is in one hand or in both hands, the carrying style of shopping bags varies from person to person depending on the weight, shape, and type of handles of the carried bags. This daily activity might be associated with risk factors contributing to the development of low back pain (LBP) due to the compression forces on the lumbar spine [1–3]. In addition, other risks such as strains and sprains in the fingers, wrists, elbows, and shoulders are present [2, 3].

Daily walking and carrying tasks cause spinal shrinkage, reduce the height of the intervertebral discs, and might produce abnormal compressive loads on the lumbar spine due to trunk muscles' contraction [4, 5]. Carrying loads causes height loss due to intervertebral discs' compression along with spinal curvature increase, which might result in LBP [3, 6, 7]. According to the Work Practices Guide for Manual Lifting published by the National Institute for Occupational Safety and Health, loads carried that resulting in developing of 6400 Newton, or more, of the compression load on the lumbosacral joint (L5/S1) are considered hazardous [8].

In general, it was reported that carrying a load in one hand poses more load on the lower back than carrying twice as much that amount but distributed evenly between both hands [9]. In addition, carrying a load in a single grocery bag held against chest is corresponding to a higher heart rate than dividing the same weight evenly in two grocery bags carried in hands on the body sides [2]. The reported research emphasizes the merit of balancing carried loads on both body sides.

Another manifestation of carrying effects is the increased ground reaction forces and plantar pressures while carrying loads compared to unloaded gait [10, 11]. As expected, biomechanical studies of walking while carrying weights have shown that vertical ground reaction forces [12], as well as plantar pressures [13], are higher when compared to walking without carrying weights. Most experimental studies of load carrying had investigated symmetrical carrying technique in a laboratory setting.

Hands, as sensitive and critical parts of the body, are involved in a significant amount of weight carrying in daily activities, which makes it necessary to protect them from injury [14]. While carrying shopping bags, shoppers may experience marks, discomfort, and/or pain in their fingers and palms caused by the exerted pressure of the relatively thin flexible plastic handles of the shopping bags. A variety of designs of plastic bag holders are commercially available to alleviate this unpleasant pain.

Researchers investigated the problem of walking while carrying loads through different approaches such as the investigation of trunk kinematics [3, 6, 7], investigation of trunk loading [9, 15], gait analysis [4, 10, 11, 16], and cardiovascular response [2]. In their approaches, researchers attempted to investigate various situations of carrying that are likely to represent real-life situations, whether it is two-hands or one-hand carrying, in front of the body or beside the body, or carrying a backpack.

To the author's knowledge, no research was conducted to investigate the influence of walking while carrying the load on trunk muscles' activities and plantar pressure with or without using plastic bags' holders. Hence, the purpose of this study was to investigate the effect of walking while carrying shopping bags of different loads in one or two hands on trunk muscles' activities and plantar pressure with or without using plastic bags' holders. In addition, the study investigated the discomfort and pain in fingers and palms while carrying these shopping bags with or without using plastic bags' holders.

## 2. Methodology

### 2.1. Experimental Design

**Figure 1:**
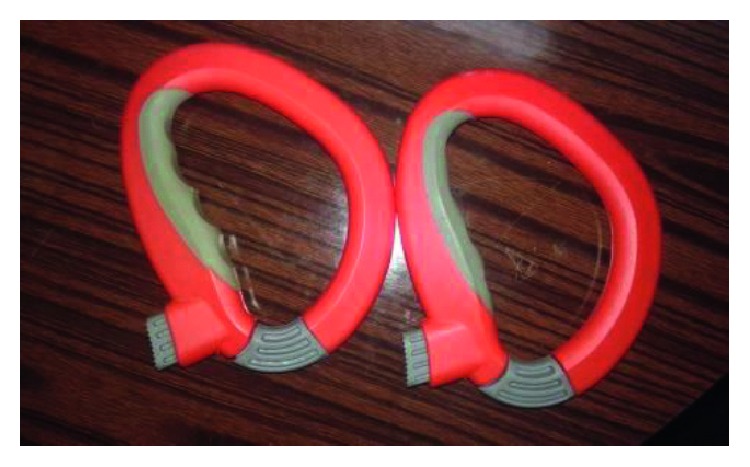
Plastic bags' holders that were employed in the experiment.

A laboratory experiment was carried out to investigate the effects of walking while carrying shopping bags in one or two hands on trunk muscles' activities and plantar pressure with or without using plastic bag holders. The experiment used a repeated measures design with three independent variables: (1) bag holding style (two levels—with or without plastic bags' holder); (2) carrying technique (two levels—dominant hand alone or two hands); and (3) shopping bags' weight (three levels—50, 100, and 150 N). Plastic bags' holders that were used in the experiment are illustrated in Figure 1. Borghols et al. [17] classified those weight levels as low and medium effort exerted by the cardiorespiratory system during active exercise. As a result, there were twelve different experimental conditions. Each shopping bag used was filled with a 25 N load (i.e., a 50 N weight consisted of two bags). Cardiac cost (CC), discomfort rating (DR), task muscle activity as a percent of the muscle activity of maximum voluntary contraction (%MVC), and peak plantar pressure (PPP) were the dependent variables.

### 2.2. Statistical Analysis

Statistical analysis was performed using the Statistical Package for the Social Sciences Software (SPSS Version 22; www.spss.com). The significance level was set to 0.05, and factors identified as having a significant effect on the dependent variables were further analyzed using Tukey's test, *t*-test, or simple effect technique to identify what levels of the factor are different in their effect on the dependent variables. In addition, if an interaction was found to have a significant effect on the dependent variables, a simple effect technique was conducted to demonstrate the effect at each level of the shopping bags' weight factor [18].

### 2.3. Participants

Thirteen young, active college male students (mean ± standard deviation [SD]: age 21 ± 1.6 years; mass 60.3 ± 7.8 kg; height 171.9 ± 5.4 cm) volunteered to participate in this study. All participants were informed the purpose of the experiment and signed consent forms (E-16-01723) before the start of the experiment. All participants were right handed. None of the participants reported a history of orthopedic injury, lower extremity trauma, deformities, or vascular diseases. Participants undertook a clinical examination to assure the absence of any hip or knee pathology that might affect participants' gait while walking. Foot examination included measurement of passive and active range of motion of the ankle joint, subtalar joint, and metatarsophalangeal joints, and pronation/supination.

### 2.4. Measured Responses and Used Equipment

#### 2.4.1. Cardiac Cost

Electrocardiogram (ECG) was recorded using Biosig Insta-Pulse heart rate monitor #BIS-203. The instrument was calibrated according to the manufacturer's procedures. Cardiac cost (CC) was calculated as the difference between the mean heart rate during the test and the heart rate when the participant stood at the start point after a three-minute period of seating [19, 20].

#### 2.4.2. Discomfort Ratings

**Figure 2:**
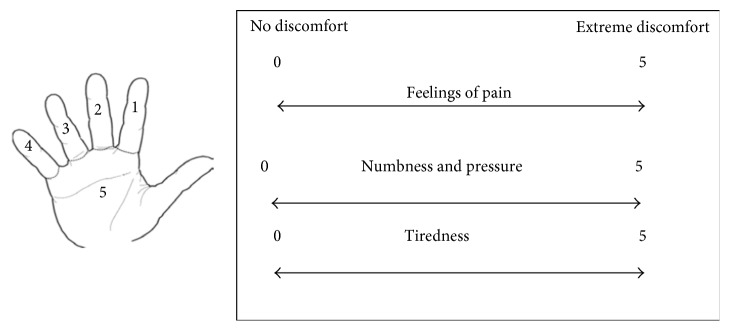
Rating scales and hand map for the subjective assessments for hand map of the index finger (1), middle finger (2), ring finger (3), little finger (4), and palm (5) and visual analogue subjective rating scales of feelings of pain (top), numbness and pressure (middle), and tiredness (bottom).

Ratings assessed locally perceived discomfort (LPD) in the hand, forearm, upper arm, and shoulder. The LPD method consisted of a detailed hand‐wrist map with five regions, as shown in Figure 2. Three 12 cm line drawings associated with each body part were shown to the participants to describe discomfort in terms of pain, numbness and pressure, and fatigue. A six-point scale was used to assess discomfort (ranging from 0 = no discomfort to 5 = extreme (almost unbearable) discomfort) in each region [21]. Each participant was asked to indicate and rate any discomfort by marking an asterisk on the specified line at the start of each trial and immediately after completing the trial. Discomfort scales are easy to use and require almost no training [22].

#### 2.4.3. Muscle Activity

**Figure 3:**
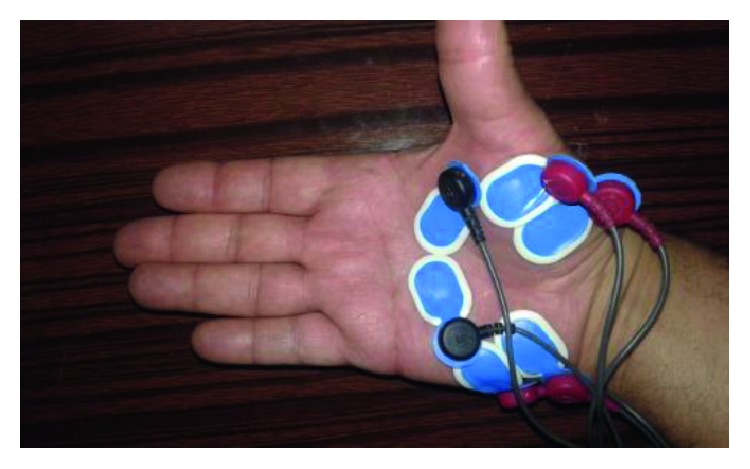
The positioning of the electrodes for the “right hypothenar” and “right thenar.”

Surface electromyogram (sEMG) was used to assess muscle activation during the carrying and walking tasks. Standardized procedures were followed to record muscles' activities in the following muscle groups [23–29]: (1) right hypothenar (RHT), (2) right thenar (RT), (3) right brachioradialis (RB), (4) right flexor digitorum superficialis (FDS), (5) right medial deltoid (RD), (6) right lower trapezius (RLT), (7) right erector spinae (RES), and (8) left erector spinae (LES). The selected muscles were involved in grasping and balancing while carrying the plastic shopping bags. Muscle activation is assumed the same between left and right sides during two-hands carrying for the muscles investigated only on the right side of the participant. The positions of the electrodes for the “right hypothenar” and “right thenar” are illustrated in Figure 3.

Muscles' activities were recorded using a wireless portable 8-channel Biomonitor ME6000 EMG (Mega Electronics Ltd., Kuopio, Finland) with a band-pass filter of bandwidth 8–500 Hz and a 14-bit A/D converter at a sampling rate of 1000 Hz [30]. Raw data were recorded and processed using MegaWin 3.1. (Mega Electronics Ltd., Kuopio, Finland) and filtered using a bidirectional fourth order, 20 Hz low-pass Butterworth filter to remove high-frequency noise from the sample [31].

Disposable Ag/AgCl Ambu Blue Sensor, Denmark, surface electrodes were used for the sEMG. Prior to installing sEMG electrodes, skin was shaved and cleaned using alcohol to minimize skin impedance to less than 20 kΩ. Electrodes were 1 cm in diameter and were placed 2 cm apart, in order to minimize potential cross talks from adjacent neutral sites. Electrodes were placed at the midpoint of the palpated muscle belly about halfway between the motor endpoint zone and the distal part of the muscle, longitudinal to the muscle fibers. All installed electrodes were affixed to the skin using strapping to minimize potential movement artifacts. No participant reported in any way that the strapping interfered with participant movement.

sEMG of isometric maximum voluntary contraction (MVC) was recorded prior to each trial (experimental condition), for each of the investigated muscles, to be used for normalizing each muscle's peak value of sEMG during the trial. Joints were placed at an appropriate angle, and all isometric actions were resisted by a chain connected to fixed horizontal climbing bars [32]. Participants were instructed to contract their muscles maximally and hold for three seconds. Isometric MVC procedures and their corresponding sEMG recording were repeated three times with rest periods of 90 seconds in between to relief muscle fatigue due to the maximal contraction [19]. Measurement procedures were standardized for body posture, verbal instructions, and encouragement [33] and were carried out by the principal investigator. The protocols of measuring the flexion MVCs of the right hypothenar and right thenar muscles were similar to those used in the study of Griffin et al. [34].

#### 2.4.4. Peak Plantar Pressure

Peak plantar pressure (PPP) is a measure of the maximum pressure or force acting on the interface between the foot and the ground while they are in contact to provide an indication of foot and ankle function during activities. A Tekscan Mat Model Strideway 2 with 0.91 meter wide by 2.60 meter long (Tekscan, Boston, MA, USA) was used to assess PPP during the stance phase as well as dynamic foot pressure during gait. Plantar pressure data were sampled at 50 Hz and passed through a PC interface board (Super-Receiver) to the computer. Then, data were made available for storage and analysis through the HR Mat System Software. The mat was calibrated for each subject using his weight before the data were collected. The reliability of the pressure-sensitive sensing system was well documented by other studies [35]. Ahroni et al. [36] reported fair to good reliability for high-pressure levels under the foot, heel, metatarsal head, and hallux.

For each experimental condition, the data from the three normal-speed walking loops were included in the data analysis to ensure stabilization of the subject's performance. A loop's data were not accepted if the subject altered his stride or visually targeted the pressure mat. The average of the three highest pressure recordings at the heel, metatarsal heads, and great toe of both feet was used for analysis. Loops' data were averaged for the statistical analysis.

### 2.5. Experimental Protocol

The experiment was performed in thirteen sessions. For the first session, the participant was asked to wear suitable light clothing (T-shirt, shorts, and light shoes). During the first session, the principal investigator performed the screening processes; the participant signed the consent form; then participant's weight and anthropometric and demographic data were collected. Each of the other twelve experimental sessions represented one experimental condition. Each participant carried out one experimental condition a day. Participants were asked not to be involved in any physical activities that could cause fatigue prior to experimental sessions. The twelve experimental sessions were randomized to minimize learning effects.

**Figure 4:**
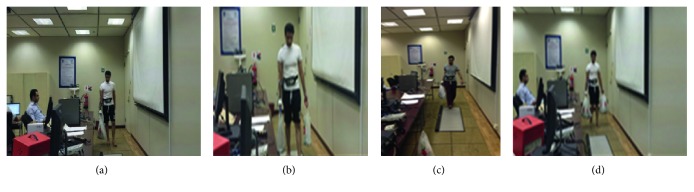
Participant carrying shopping bags (a) in one hand with shopping bags' holder; (b) in two hands with shopping bags' holders; (c) in one hand without shopping bags' holder; and (d) in two hands without shopping bags' holders.

At the beginning of each experimental session, the sEMG of a participant's MVCs was recorded; then the participant was asked to sit on a chair at the starting point of the walkway for 3 minutes to stabilize his physiological parameters. Resting heart rate and discomfort rating data were collected each time prior to walking while the participant was standing. Then, the participant was asked to carry the plastic shopping bags assigned to the experimental condition and walk for 5 minutes around the inside perimeter of the Ergonomics Laboratory, as shown in Figure 4. The round track was about 14 m long and 7 m wide with a perimeter of 42 meters.

Participants were asked to walk steadily at their normal walking speed and avoid any sudden or aggressive moves that might result in sudden loading of the spine [3, 37–39]. After 90 seconds of walking, PPPs and 15-second sEMG muscular activities were recorded one time every minute for three minutes. Walked distance during the 5 minutes was measured, and walking speed was calculated. Heart rate was recorded in the last minute of the 5-minute walk. Participants were asked to rate discomfort in their hand(s) using the described scales at the end of each 5-minute walk. Average of the three PPPs was computed for the statistical analysis. Average of the three peak values of sEMG recorded during an experimental session was calculated and normalized as a percentage of the sEMG of the maximum voluntary contraction (%MVC).

## 3. Results

### 3.1. Cardiac Cost

**Figure 5:**
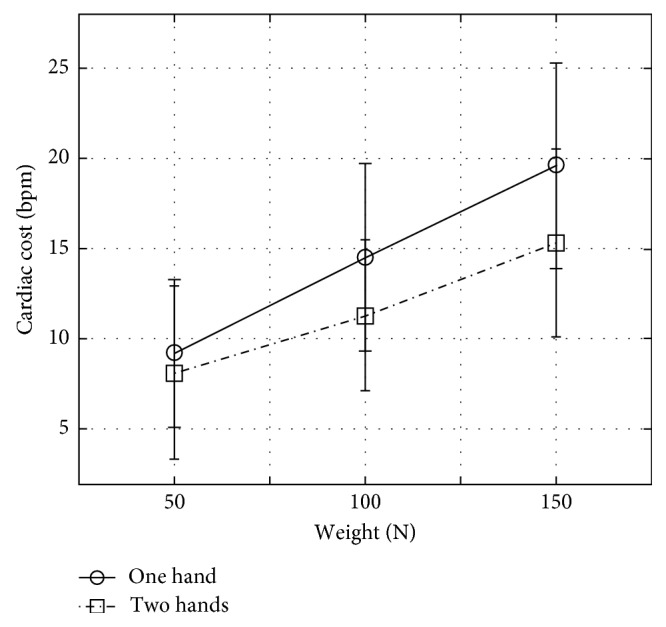
Effect of interaction between carrying technique and shopping bags' weight on the participants' cardiac costs.

Bags holding style had significant effect on cardiac cost: *F*(1, 12) = 6.917, *p* < 0.022. The cardiac cost for walking while carrying shopping bags is significantly less when using holders (mean = 13.1, SD = 3.8) than when not using holders (mean = 16.7, SD = 4.6). In addition, the two-way interaction between carrying technique and shopping bags' weight had a significant effect on cardiac cost: *F*(2, 24) = 4.149, *p* < 0.028. The cardiac cost for walking while carrying the 100 N and 150 N bags was significantly less when using two hands than when using one hand (*p* < 0.011 and *p* < 0.001, resp.); Figure 5 represents these results.

### 3.2. Discomfort Ratings

**Figure 6:**
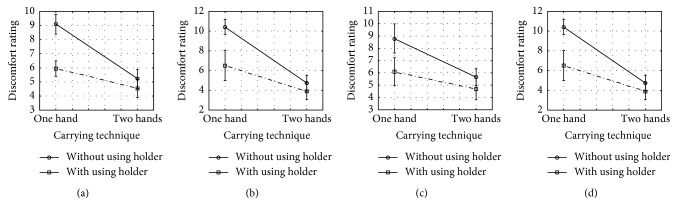
Effects of handling style by carrying method interactions on participants' discomfort ratings at (a) index finger, (b) middle finger, (c) ring finger, and (d) little finger.

The main effects of the three independent variables were significant on discomfort rating. Bag holding style had a significant effect on perceived discomfort ratings in the palm, the upper arm, and the shoulder. Carrying technique had a significant effect on perceived discomfort ratings in the palm, the upper arm, and the shoulder. Shopping bags' weight had a significant effect on perceived discomfort ratings in the index finger, the middle finger, the ring finger, the little finger, the palm, the upper arm, and the shoulder. The two-way interaction between the holding style and the carrying technique had significant effect on perceived discomfort ratings in the index finger, the middle finger, the ring finger, and the little finger. Higher discomfort rating was associated with carrying bags in one hand without using the holder compared to carrying using the holder. All *F* and *p* values are listed in Table 1, and interaction plots are represented in Figure 6.

### 3.3. sEMG Muscle Activities

Only the two-way interaction between the carrying technique and the shopping bags' weight had a significant effect on the %MVC of the hypothenar, thenar, brachioradialis, flexor digitorum superficialis, medial deltoid, lower trapezius, and left erector spinae. All *F* and *p* values are listed in Table 2.

**Figure 7:**
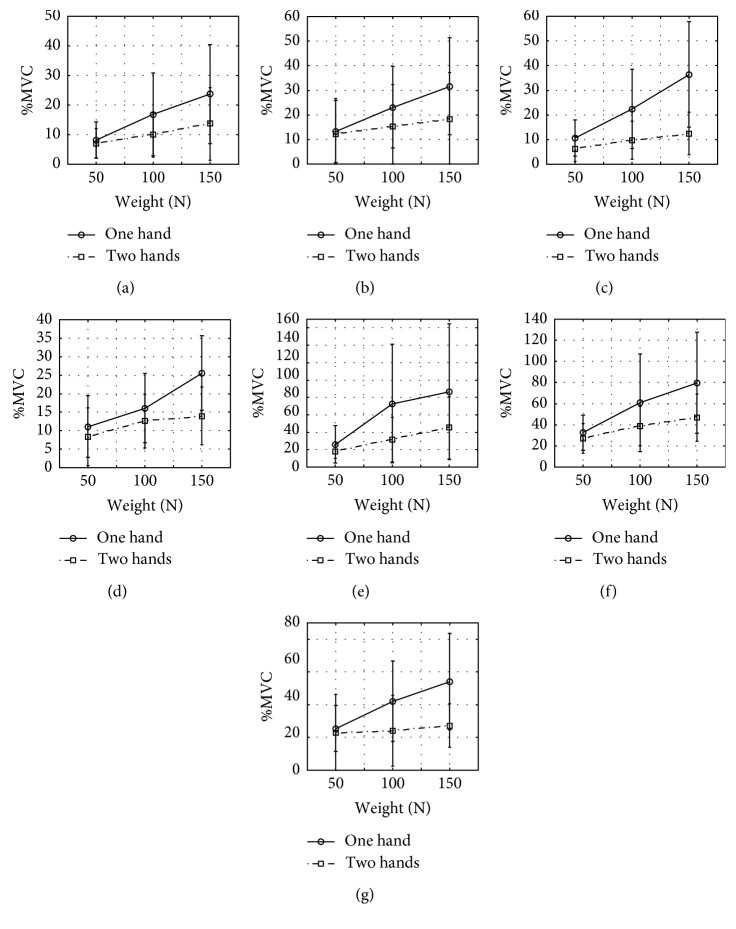
Effects of the two-way interaction between the carrying technique and the shopping bags' weight on the participants' %MVC of (a) RHT, (b) RT, (c) RB, (d) FDS, (e) RD, (f) LT, and (g) LES muscles.

At 100 N, %MVC was significantly lower when both hands were used to carry grocery bags compared to carrying with only one hand in hypothenar, thenar, brachioradialis, medial deltoid, lower trapezius, and left erector spinae muscles. At 150 N, %MVC was significantly lower when both hands were used to carry grocery bags compared to using only one hand in hypothenar, thenar, brachioradialis, flexor digitorum superficialis, medial deltoid, lower trapezius, and left erector spinae muscles. All interaction plots are represented in Figure 7.

### 3.4. Peak Plantar Pressure (PPP)

**Figure 8:**
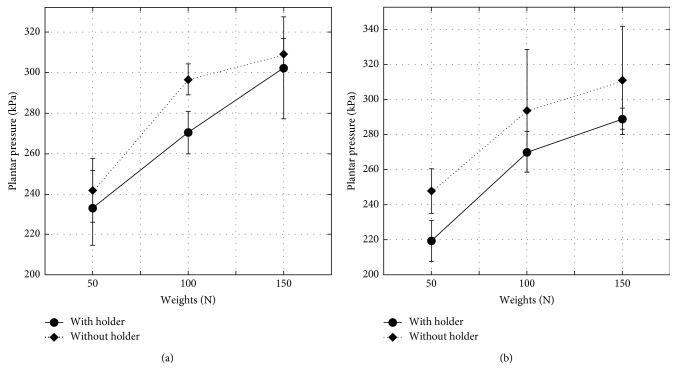
Effects of the three-way interaction among holding style, carrying technique and shopping bags' weight on the participants' peak plantar pressures: (a) one hand; (b) two hands.

Only the three-way interaction among holding style, carrying technique, and shopping bags' weight had a significant effect on the PPP: *F*(2, 24) = 3.754, *p* < 0.033. A simple effect technique was used to analyze the higher level of interaction. For the one-hand load carrying, PPPs were significantly lower when the bags' holder was used than when it was not used at the 50 N load (*p* < 0.031) and the 100 N load (*p* < 0.0001). For the two-hand load carrying, PPPs were significantly lower when the bags' holder was used than when it was not used at the 50 N load (*p* < 0.0001), the 100 N load (*p* < 0.005), and the 150 N load (*p* < 0.008). Interaction plots are represented in Figure 8.

## 4. Discussion and Conclusions

The purpose of this study was to investigate the effect of walking while carrying shopping bags of different loads in one or two hands on trunk muscles' activities and plantar pressure with or without using plastic bags' holders. In addition, the study investigated the discomfort and pain in fingers and palms while carrying these shopping bags with or without using plastic bags' holders. The motivation for this study was the constant need for improving human performance and reducing pain and possibilities of injuries while performing simple, inevitable daily activities such as walking and carrying plastic shopping bags.

It is possible to improve the performance of the carrying shopping bags task by simply conserving associated energy expenditure through the use of carrying assistive equipment that would alleviate associated stresses and discomfort. Carrying grocery bags might be achieved by one of the two essential techniques, either holding a load close to one side of the body or holding loads close to both sides of the body.

In general, the task of walking and carrying loads has been approached by researchers with the purpose of investigation and description of its effects on the human body. None of these approaches considered the walking while carrying plastic shopping bags with or without bags' holder. In addition, the literature does not provide a clear answer for which technique produces less cardiovascular cost, less discomfort, and less musculoskeletal stress.

This study found less cardiac costs associated with the two-hands-on-the-sides carrying technique compared to the one-hand carrying technique, especially with heavier shopping bags. The results obtained in this study were compared with the results from Fredericks et al. [40] and Irion et al. [2]. Carrying shopping plastic bags using holders allowed participants to carry more loads on both body sides comfortably than carrying one load on one body side.

The study found an evidence of favoring the two-hands carrying to the one-hand carrying in general based on the investigated physiological responses in the form of lower cardiac cost and lower %MVC and subjective response in the form of lower discomfort rating. This finding is in support of other studies, which investigated different responses such as trunk kinematics [3, 6, 7], trunk loading [8, 15], and gait analysis [4, 10, 11, 16].

Carrying shopping bags using the holder decreased both discomfort ratings and PPPs when compared to carrying them without using holders. This finding implies that the holder volume and shape reduced the stress on the palm and the fingers by distributing the bag load over a larger skin area. In addition, it allowed more control of the carried bags, which improved the participants' whole body posture while walking resulting in a lower PPP.

This study examined the responses of healthy young males when carrying grocery bags weighing approximately 50, 100, and 150 N while walking at their normal walking speed for 5 min. The loads and walking duration were chosen to create an intensity of exertion ranging from light to somewhat hard for this young male population, based on the assumption that the average person would be willing to experience this range of exertion during grocery carrying [2]. The results of this research can only point out the differences in cardiovascular stresses related to kinesiology principles that may apply to different techniques of carrying. This study used loads representative of actual grocery purchases to promote one carrying technique as a means of energy conservation and comfort for all users. Finally, it is useful to carry grocery bags close to the body with both hands using holders that are available in the local market.

Future research might build on the findings of this study and extend to investigate other populations, especially the young females and the elderly people (both males and females) as these two populations are heavily involved in the task of shopping and carrying shopping bags possibly for periods longer than 5 minutes.

## Figures and Tables

**Table 1:** *F* and *p* values for the upper extremity parts studied. Their discomfort rating was significantly affected by the main effects of the three independent variables and the interaction between the handling style and the shopping bags' weight.

	Weight		Holding style		Carrying technique		Holding style × carrying technique	
	*F*(1, 12)	*p*	*F*(1, 12)	*p*	*F*(1, 12)	*p*	*F*(1, 12)	*p*
Index finger	214.307	<0.0001	—	—	—	—	15.421	<0.002
Middle finger	188.033	<0.0001	—	—	—	—	7.861	<0.016
Ring finger	118.403	<0.0001	—	—	—	—	7.885	<0.016
Little finger	84.112	<0.0001	—	—	—	—	14.186	<0.003
Palm	180.746	<0.0001	6.973	<0.022	38.167	<0.0001	—	—
Upper arm	123.29	<0.0001	76.134	<0.0001	22.29	<0.0001	—	—
Shoulder	115.746	<0.0001	109.02	<0.0001	13.883	<0.003	—	—

**Table 2:** *F* and *p* values for the muscles studied. Their %MVC was significantly affected by the two-way interaction between the carrying technique and the shopping bags' weight.

	Weight × carrying technique	
	*F*(2, 24)	*p*
Hypothenar	8.682	<0.001
Thenar	22.085	<0.0001
Brachioradialis	22.667	<0.0001
Flexor digitorum superficialis	13.804	<0.0001
Medial deltoid	3.831	<0.041
Lower trapezius	9.001	<0.001
Left erector spinae	27.838	<0.0001

## References

[1] Kostova V., Koleva M. (2001). Back disorders (low back pain, cervicobrachial and lumbosacral radicular syndromes) and some related risk factors. *Journal of the Neurological Sciences*.

[2] Irion G., Melancon H. H., Nuchereno N., Strawbridge B., Young J. (2010). Cardiovascular responses to carrying groceries in bags with and without handles. *Journal of Acute Care Physical Therapy*.

[3] Watson H., Simpson A., Riches P. E. (2012). The effects of upper limb loading on spinal shrinkage during treadmill walking. *European Spine Journal*.

[4] Park H., Branson D., Kim S. (2014). Effect of armor and carrying load on body balance and leg muscle function. *Gait and Posture*.

[5] Dunlop R. B., Adams M. A., Hutton W. C. (1984). Disc space narrowing and the lumbar facet joints. *Journal of Bone and Joint Surgery*.

[6] Fowler N. E., Rodacki A. L. F., Rodacki C. D. (2006). Changes in stature and spine kinematics during a loaded walking task. *Gait and Posture*.

[7] Goode J. B., Theodore B. M. (1983). Voluntary and diurnal variation in height and associated changes in spinal curves. *Engineering in Medicine*.

[8] NIOSH (1981). *Work Practices Guide for Manual Lifting (No. 81-122)*.

[9] McGill S. M., Marshall L., Andersen J. (2013). Low back loads while walking and carrying: comparing the load carrying in one hand or in both hands. *Ergonomics*.

[10] Castro M., Abreu S., Sousa H., Machado L., Santos R., Vilas-Boas J. P. (2013). Ground reaction forces and plantar pressure distribution during occasional loaded gait. *Applied Ergonomics*.

[11] Pau M., Corona F., Leban B., Pau M. (2011). Effects of backpack carriage on foot-ground relationship in children during upright stance. *Gait and Posture*.

[12] Willson J., Torry M. R., Decker M. J., Kernozek T., Steadman J. R. (2000). Effects of walking poles on lower extremity gait mechanics. *Medicine and Science in Sports and Exercise*.

[13] Hudson D. (2013). The effect of walking with poles on the distribution of plantar pressures in normal subjects. *PM&R*.

[14] Chung M. K., Lee I., Kee D. (2005). Quantitative postural load assessment for whole body manual tasks based on perceived discomfort. *Ergonomics*.

[15] Subramaniyam M., Min S. N., Park S. J., Park S. (2015). Muscle activity and spinal loading in lifting symmetrical loads beside the body compared to in front of the body. *Journal of Mechanical Science and Technology*.

[16] LaFiandra M., Wagenaar R. C., Holt K. G., Obusek J. P. (2003). How do load carriage and walking speed influence trunk coordination and stride parameters?. *Journal of Biomechanics*.

[17] Borghols E. A., Dresen M. H., Hollander A. P. (1978). Influence of heavy weight carrying on the cardiorespiratory system during exercise. *European Journal of Applied Physiology and Occupational Physiology*.

[18] Keppel G. (1982). *Design and Analysis: A researcher’s Handbook*.

[19] Ramadan M. Z., Al-Shayea A. M. (2013). A modified backpack design for male school children. *International Journal of Industrial Ergonomics*.

[20] Pierret B., Desbrosses K., Paysant J., Meyer J. (2014). Cardio-respiratory and subjective strains sustained by paraplegic subjects, when travelling on a cross slope in a manual wheelchair (MWC). *Applied Ergonomics*.

[21] Groenesteijn L., Eikhout S. M., Vink P. (2004). One set of pliers for more tasks in installation work: the effects on (dis)comfort and productivity. *Applied Ergonomics*.

[22] Drury C. G., Coury B. G. (1982). A methodology for chair evaluations. *Applied Ergonomics*.

[23] Brandsm J. W., Schreuders T. A. R. (2001). Sensible manual muscle strength testing to evaluate and monitor strength of the intrinsic muscles of the hand: a commentary. *Journal of Hand Therapy*.

[24] Kofler M., Kumru H., Stetkarova I., Schindler C., Fuhr P. (2007). Muscle force up to 50% of maximum does not affect cutaneous silent periods in thenar muscles. *Clinical Neurophysiology*.

[25] Waters R. L., Stark L. Z., Gubernick I., Bellman H., Barnes G. (1990). Electromyographic analysis of brachioradialis to flexor pollicis longus tendon transfer in quadriplegia. *Journal of Hand Surgery*.

[26] Popp W. L., Lambercy O., Müller C., Gassert R. (2016). Effect of handle design on movement dynamics and muscle co-activation in a wrist flexion task. *International Journal of Industrial Ergonomics*.

[27] Hodder J. N., La Delfa N. J., Potvin J. R. (2016). Testing the assumption in ergonomics software that overall shoulder strength can be accurately calculated by treating orthopedic axes as independent. *Journal of Electromyography and Kinesiology*.

[28] Ekstrom R. A., Soderberg G. L., Donatelli R. A. (2005). Normalization procedures using maximum voluntary isometric contractions for the serratus anterior and trapezius muscles during surface EMG analysis. *Journal of Electromyography and Kinesiology*.

[29] Jakobsen M., Sundstrup E., Andersen C., Aagaard P., Andersen L. (2013). Muscle activity during leg strengthening exercise using free weights and elastic resistance: Effects of ballistic vs. controlled contractions. *Human Movement Science*.

[30] Burhan N., Kasno M., Ghazali R., Said R., Abdullah S., Jali M. (2017). Analysis of the biceps brachii muscle by varying the arm movement level and load resistance band. *Journal of Healthcare Engineering*.

[31] Robertson D., Dowling J. (2003). Design and responses of Butterworth and critically damped digital filters. *Journal of Electromyography and Kinesiology*.

[32] Hintermeister R. A., Lange G. A., Schultheis J. M., Bey M. J., Hawkings R. J. (1998). Electromyographic activity and applied load during shoulder rehabilitation exercises using elastic resistance. *American Journal of Sports Medicine*.

[33] Chen B., Bates B. T. (2000). Comparing of F-scan in-sole and AMTI force plate system in measuring vertical ground reaction force during gait. *Physiotherapy Theory and Practice*.

[34] Griffin L., Jun B. G., Covington C., Doucet B. M. (2008). Force output during fatigue with progressively increasing stimulation frequency. *Journal of Electromyography and Kinesiology*.

[35] Hsiao H., Guan J., Weatherly M. (2002). Accuracy and precision of two in-shoe pressure measurement systems. *Ergonomics*.

[36] Ahroni J. H., Boyko E. J., Forsberg R. (1998). Reliability of F-scan in-shoe measurements of plantar pressure. *Foot & Ankle International*.

[37] Watanabe K., Asaka T., Wang Y. Effects of backpack load and gait speed on plantar force during treadmill walking.

[38] Goffar S. L., Reber R. J., Christiansen B. C., Miller R. B., Naylor J. A., Rodriguez B. (2013). Changes in dynamic plantar pressure during loaded gait. *Physical Therapy*.

[39] Zhou J., Dai B., Ning X. (2013). The assessment of material handling strategies in dealing with sudden loading: influences of foot placement on trunk biomechanics. *Ergonomics*.

[40] Fredericks T. K., Fernandez J. E., Rodriques C. C. (1994). Psychophysically acceptable weights for a combination lifting task using bags with handles. *Journal of Human Ergology*.

